# Complement-Mediated Differential Immune Response of Human Macrophages to *Sporothrix* Species Through Interaction With Their Cell Wall Peptidorhamnomannans

**DOI:** 10.3389/fimmu.2021.749074

**Published:** 2021-11-15

**Authors:** Gabriela W. P. Neves, Sarah Sze Wah Wong, Vishukumar Aimanianda, Catherine Simenel, J. Iñaki Guijarro, Catriona Walls, Janet A. Willment, Neil A. R. Gow, Carol A. Munro, Gordon D. Brown, Leila M. Lopes-Bezerra

**Affiliations:** ^1^ Cell Biology Department, Rio de Janeiro State University, Rio de Janeiro, Brazil; ^2^ Institut Pasteur, Molecular Mycology Unit, CNRS UMR2000, Paris, France; ^3^ Institut Pasteur, Biological NMR and HDX-MS Technological Platform, CNRS UMR3528, Paris, France; ^4^ Aberdeen Fungal Group, Institute of Medical Sciences, University of Aberdeen, Aberdeen, United Kingdom; ^5^ Medical Research Council Centre for Medical Mycology at the University of Exeter, Exeter, United Kingdom; ^6^ Biomedical Institute and Technology and Innovation Center (CIETEC), São Paulo University, São Paulo, Brazil

**Keywords:** *Sporothrix schenckii*, *Sporothrix brasiliensis*, complement receptor-3 (CR3), cell wall, peptidorhamnomannan (PRM), human macrophages, innate immune response

## Abstract

In this study, the human immune response mechanisms against *Sporothrix brasiliensis* and *Sporothrix schenckii*, two causative agents of human and animal sporotrichosis, were investigated. The interaction of *S. brasiliensis* and *S. schenckii* with human monocyte-derived macrophages (hMDMs) was shown to be dependent on the thermolabile serum complement protein C3, which facilitated the phagocytosis of *Sporothrix* yeast cells through opsonization. The peptidorhamnomannan (PRM) component of the cell walls of these two *Sporothrix* yeasts was found to be one of their surfaces exposed pathogen-associated molecular pattern (PAMP), leading to activation of the complement system and deposition of C3b on the *Sporothrix* yeast surfaces. PRM also showed direct interaction with CD11b, the specific component of the complement receptor-3 (CR3). Furthermore, the blockade of CR3 specifically impacted the interleukin (IL)-1β secretion by hMDM in response to both *S. brasiliensis* and *S. schenckii*, suggesting that the host complement system plays an essential role in the inflammatory immune response against these *Sporothrix* species. Nevertheless, the structural differences in the PRMs of the two *Sporothrix* species, as revealed by NMR, were related to the differences observed in the host complement activation pathways. Together, this work reports a new PAMP of the cell surface of pathogenic fungi playing a role through the activation of complement system and *via* CR3 receptor mediating an inflammatory response to *Sporothrix* species.

## Introduction

The fungal genus *Sporothrix* harbors thermo-dimorphic species that often cause sporotrichosis, a sapronosis and/or an anthropozoonotic disease that can be transmitted from cats to humans (1). The yeast phase of *Sporothrix* species is the parasitic morphotype, which grows *in vitro* at 37°C (1). For more than a century, sporotrichosis was considered a benign subcutaneous mycosis caused only by *Sporothrix schenckii*. However, this paradigm has changed due to the discovery of new pathogenic cryptic species, *S. schenckii sensu stricto*, *Sporothrix brasiliensis*, and *Sporothrix globosa*, a pathogenic clade within the *Sporothrix* genus (2, 3). Among the new pathogenic species, *S. brasiliensis* has the higher clinical-epidemiological impact (4, 5) as the main species related to cat to human transmission in South America (6–8), while *S. schenckii* has a worldwide distribution (5). In addition, *S. brasiliensis* is the prevalent species related to sporotrichosis in cats (*Felis catus domesticus*) followed by *S. schenckii* (9, 10).

*S. brasiliensis* shows differences in its pathogenicity compared to other pathogenic species (11, 12). The infection due to *S. brasiliensis* is characterized by serious clinical features in animals as well as in the human host (13–17). While *S. brasiliensis* exhibits a high virulent profile, the older known pathogenic species *S. schenckii* causes a benign chronic subcutaneous mycosis in humans and a moderate virulence profile in animals (11, 12). Host innate immune system plays a crucial role against invading microbes; however, the mechanism implied by the innate immune system against *Sporothrix* is ambiguous. Neutrophils and macrophages from chronic granulomatous diseased (CGD) mice lacking the capacity to produce microbicidal reactive oxygen intermediate (ROI) failed to control the growth of *S. schenckii*; moreover, CGD mice were susceptible to disseminated systemic infection upon subcutaneous inoculation (18). In humans, however, *S. schenckii* could be eliminated by polymorphonuclear cells through H_2_O_2_-KI-myeloperoxidase but was resistant to killing by neutrophil-generated H_2_O_2_ (19, 20). The pathogen-associated molecular patterns (PAMPs) and the molecular mechanisms involved in the host response are crucial for the comprehension of the immunopathology of sporotrichosis (21). Indeed, the interaction between the PAMPs and the host pathogen-recognition receptors (PRRs) is key for fungal recognition and clearance by phagocytes, the cellular components of the innate immune system (22). The Toll-like-receptor 4 (TLR4) has gained prominence as a PRR in recognizing and mounting a protective immune response against *S. schenckii* and *S. brasiliensis* (23–25). Besides TLR4, TLR2, mannose receptor (MR), and Dectin-1 have been addressed: (i) TLR2 was described as important for the phagocytosis of the yeast form of *S. schenckii* by murine macrophages (26) and plays a role in *S. brasiliensis* infection (27); (ii) MR was involved in the sensing of *S. schenckii* conidia by THP-1 macrophages (28) and in the immune response by human peripheral blood mononuclear cells (PBMCs) challenged with yeast/conidial morphotypes of both *S. schenckii* and *S. brasiliensis* (25); and (iii) Dectin-1 was described as a key receptor for the cytokine stimulation of human PBMCs infected with conidia, germlings, and yeast morphotypes of *S. schenckii* or the yeast morphotype of *S. brasiliensis* (25). Despite the recognition of these host immune cell surface PRRs, the PAMPs and the molecular mechanisms involved in the recognition of these two *Sporothrix* species, *S. schenckii* and *S. brasiliensis*, remained poorly understood.

The fungal cells are endowed with a cell wall that protects and provides mechanical strength. The fungus cell wall is considered an extracellular structure/organelle that is the first to come into contact with the host’s immune system and, in addition, harbors important PAMPs (22, 29). The composition of the cell wall may be common and/or distinct among the fungal species belonging to one genus (22, 29). In consequence, differences on the fungal cell wall structure of pathogenic fungi are closely related to their recognition by distinct PRRs of macrophages, neutrophils, and/or dendritic cells and, as a result of this interaction, modulate the innate immune response (22, 30). In *Sporothrix* species, a cell wall model has been proposed recently showing that the major cell wall component present on the outer layer of both *S. schenckii* and *S. brasiliensis* is a peptidorhamnomannan (PRM) (31). The PRM component has several structural characteristics in common between these two species but also has a unique organization in *S. brasiliensis* as shown by biochemical data and high-power field-transmission electron microscopy (31). In this study, we report for the first time that the complement receptor-3 (CR3) expressed by the human macrophages recognizes PRMs and mounts a protective inflammatory response against these two *Sporothrix* species. Furthermore, we establish by NMR the differences in structure of PRMs from these two *Sporothrix* species, which are associated with the activation of the complement system through different pathways.

## Materials and Methods

### *Sporothrix* Strains and Culture Conditions

The *Sporothrix* strains used in our study are *S. schenckii* (ATCC MYA 4820) and *S. brasiliensis* (ATCC MYA 4823), both clinical isolates from the same geographical hyperendemic region, in Rio de Janeiro state, Brazil. The virulence profile for these clinical isolates has been determined (11), and their cell wall structures were well characterized (31). To obtain them in yeast form, both the strains were grown in YPD broth (pH 7.8; Difco, Detroit, MI, USA) for 4 days at 37°C under orbital agitation, as previously described (25, 31). The yeasts cells were collected, passed through a Falcon filter (40 µm) to remove any hyphal fragments and/or yeast clusters (31), washed twice in Dulbecco’s modified Eagle’s medium (DMEM) (LGC Biotecnologia, SP, Brazil), and counted in a hemocytometer. For the interaction assays, the yeast cell concentration was adjusted accordingly.

### Preparation of Human Monocyte-Derived Macrophages

Blood samples were collected from cubital veins of eight healthy volunteers, and human monocyte-derived macrophages were generated as described previously (32, 33). All volunteers gave informed consent prior to their participating in this study. The number of the Certificate of Presentation for Ethical Consideration related to this study is 62785716.2.0000.5259. Briefly, peripheral venous blood was collected in ethylenediamine tetraacetic acid (EDTA)-coated tubes, pooled for each donor, and diluted in Hanks’ balanced salt solution (HBSS, Cultilab, SP, Brazil). Lymphoprep (Axis-Shield, Oslo, Norway) gradient fractionation was used to separate PBMCs from the whole blood. Isolated PBMCs were then washed with HBSS and resuspended in DMEM (LGC Biotecnologia, SP, Brazil). To isolate monocyte subpopulation from PBMCs, a positive selection was performed using human CD14+ microbeads (Miltenyi Biotec, Bergisch Gladbach, Germany). In parallel, sera were separated from clotted blood samples of each donor (whole serum samples); part of the sera was heat-inactivated at 56°C for 30 min (complement-inactivated serum samples). The purified CD14+ monocytes were then suspended in DMEM supplemented with 1% Pen/Strep/Glut (10,000 unit/ml penicillin; 10,000 µg/ml streptomycin; 29.2 mg/ml glutamine) solution (GIBCO^®^, NT, USA) and 10% (v/v) of autologous serum samples. These cells were seeded in culture plates and maintained for 7 days at 37°C in an atmosphere of 5% CO_2_ for differentiation into macrophages (hMDMs).

### *Sporothrix*–Human Monocyte-Derived Macrophage Interaction Study

The hMDMs were inoculated with yeast cells of *S. schenckii* or *S. brasiliensis*. All interaction assays were performed in a 5% CO_2_ incubator at 37°C. The culture medium used was DMEM supplemented with 1% (v/v) of Pen/Strep/Glut and 10% (v/v) of either autologous whole human serum (wHS) or autologous inactivated human serum (iHS). The multiplicity of infection (MOI) of *Sporothrix*:hMDM was 3:1 or 5:1, as indicated for each experiment.

### Fluorescein Isothiocyanate Labeling of *S. schenckii* and *S. brasiliensis*

For Fluorescence-activated cell sorting (FACS) analysis and live microscopy, live yeasts were stained for 20 min at ambient temperature in the dark with 1 mg/ml fluorescein isothiocyanate (FITC; Sigma, Dorset, UK) in 0.05 M carbonate-bicarbonate buffer pH 9.6 (BDH Chemicals, VWR International, Leicestershire, UK). Following incubation, yeasts were washed with phosphate-buffered saline (PBS; 3×) to remove excess FITC and resuspended in PBS for a final suspension concentration of 10^8^ FITC-labeled yeasts/ml.

### Live-Cell Video Microscopy for Phagocytosis Assay

Assays were performed using a standard protocol with modifications (33, 34). hMDMs were washed in PBS, resuspended in prewarmed supplemented serum-free CO_2_-independent medium containing LysoTracker red DND-99 (1 µM; Invitrogen, Paisley, UK), and used immediately for the phagocytosis assays. Live FITC-labeled *S. schenckii* and *S. brasiliensis* yeast cells at 3:1 MOI were added to 10^6^ LysoTracker red DND-99-stained hMDMs seeded in a sterile glass-based imaging slide chamber of eight wells and cocultured in CO_2_-independent medium supplemented with 10% of whole human serum. 3i-Live Cell Imaging Systems featuring Yokogawa CSU-X1 (Spinning Disk Confocal Microscope coupled with an EMCCD camera, PerkinElmer, USA) was performed at 37°C in the microscope chamber. Images were captured at 1-min intervals over up to 18 h. The time lapse to start image capture (time zero) was 5 min. The Volocity^®^ 6.2 imaging analysis software was used to track phagocyte migration and yeasts uptake at 1-min intervals throughout the phagocytosis duration of 30 min or overnight (18 h).

### Flow Cytometry Analysis

*S. schenckii* and *S. brasiliensis* were analyzed for their interaction with hMDM in the presence of wHS or heat-inactivated human serum (iHS) as well as to evaluate phagocytic index upon blocking CR3, TLRs and Dectin-1. To block CR3, TLR2, TLR4, and Dectin-1 receptors, monoclonal anti-CD11b (Thermo Fisher, MA5-16528), anti-TLR2 (Abcam, ab16894), anti-TLR4 (Abcam, ab22048), and anti-Dectin-1 antibodies (35) were used, respectively. Briefly, hMDMs were preincubated with fresh DMEM medium supplemented with 10% of wHS for 30 min at 37°C in an atmosphere of 5% of CO_2_. Monoclonal antibodies were then added in a final concentration of 10 µg/ml and incubated further at 8°C for 1 h. Then, hMDMs were infected with FITC-labeled yeasts of *S. schenckii* or *S. brasiliensis*, and the interaction was allowed for a duration of 1 h at 37°C in a CO_2_ incubator. The hMDMs were washed one time with PBS (GIBCO^®^, NY, USA) pH 7.4 and incubated for 15 min at ambient temperature with Trypan blue (GIBCO^®^, NY, USA) at a final concentration of 1.2 mg/ml. The cells were washed twice with PBS and fixed with 1% *p*-formaldehyde for 15 min at ambient temperature. Finally, hMDMs were washed once with PBS and, with the aid of a cell scraper, were gently released from the wells, suspended in PBS supplemented with 3% fetal bovine serum, and analyzed in a BD FACS-Canto™ II.

### Phagocytosis Assay Using Light Microscopy

To perform kinetics assay of phagocytosis with image analysis, hMDMs were cultivated on circular coverslips in a 24-well culture plate (2.5 × 10^5^ hMDMs per well) and infected with *S. schenckii* or *S. brasiliensis* yeasts at MOI 3:1. The interaction assay was performed for 1, 4, and 18 h, and the culture medium was supplemented with wHS or iHS. After each interaction time, the number of yeasts inside the macrophages was counted (defined when a clear halo was observed around the yeast). At least 10 microscopy fields were imaged, and a minimum of 50 hMDMs were counted. Photomicrographs were done in the ×100 optical magnitude. This experiment was performed two times, each time in duplicate.

### Cytokine Determination by ELISA

To determine cytokine and long pentraxin 3 (PTX3) secretions, hMDMs were cultivated in a 96-well culture plate (0.5 × 10^5^ hMDMs per well), infected with *S. schenckii* or *S. brasiliensis* (MOI, 5:1). The interaction assay was performed for 4 or 18 h. At these time points, the culture plates were centrifuged (200 g, 15 min, 4°C) to remove cell debris. The culture supernatants were collected, used immediately, or stored at -80°C until use. PTX3 secretion was determined by Quantikine ELISA Pentraxin kit (R&D Systems). Tumor necrosis factor (TNF)-α, interleukin (IL)-1β, and IL-10 concentrations in the culture supernatants were determined by ELISA using kits (R&D Systems^®^, USA) according to the manufacturer’s instructions. Non-infected hMDM was used as a negative control, and hMDM incubated with lipopolysaccharide (LPS; 10 ng/µl; Sigma-Aldrich^®^, Brazil) was used as a positive control. CR3-, TLR2-, or TLR4-blocked hMDMs were also infected with *S. schenckii* or *S. brasiliensis* for 18 h. TNF-α and IL-1β concentrations in the culture supernatants were determined by ELISA. Non-infected hMDMs were used as non-infected control; hMDMs infected with *Sporothrix* species in the presence of wHS were used as the infected controls. Experiments were performed two/three times in duplicate (n = 4 or 6).

### Cytotoxicity by Lactate Dehydrogenase Assay

The cytotoxicity assay was performed in 96-well culture plates seeded with 0.5 × 10^5^ hMDMs/well, infected with *Sporothrix* yeasts at MOI 5:1; the interaction was arrested after 4–18 h. After each time point, the culture supernatant was collected, used immediately, or stored at -80°C until use. The lactate dehydrogenase (LDH) activity was measured using CytoTox^®^ Non-Radioactive Cytotoxicity Assay (Promega) according to the manufacturer’s instructions. This assay was performed two times in duplicate/triplicate (n = 5).

### Complement Activation

Direct C3b binding to *Sporothrix* yeast surfaces was checked by immunofluorescence labeling. Briefly, *Sporothrix* yeasts were fixed with 2.5% paraformaldehyde (PFA) at ambient temperature for 1 h and then overnight at 4°C. After quenching with 0.1 M NH_4_Cl, fixed *Sporothrix* yeasts were blocked with PBS-BSA (1%) at ambient temperature for 1 h, incubated with recombinant C3b [5 μg/ml in GVB+ (preparation that is explained below); Merck Millipore], added with anti-C3b antibodies (5 μg/ml in PBS-BSA; mouse monoclonal, MA1-40155, Invitrogen), incubated for 1 h, washed with PBS (3×), incubated with tetramethylrhodamine (TRITC)-conjugated mouse immunoglobulin G (IgG; Sigma) in the dark for 1 h, washed with PBS (3×), mounted on slides, and observed for fluorescent labeling under confocal microscope (LSM700, ZEISS).

PRMs obtained from both *Sporothrix* species as described previously (36) were dissolved at 50 µg/ml in sodium bicarbonate buffer (50 mM, pH 9.6). The 96-well microtiter plate was coated with PRM solution (100 μl/well) overnight at ambient temperature. The supernatants were discarded, and the wells were washed once in wash buffer (PBS supplemented with 0.05% Tween-20). Wells were then blocked with PBS containing 1% BSA (PBS-BSA) at ambient temperature for 1 h and washed once with wash buffer. Gelatin veronal buffer (GVB-) was prepared with 5 mM barbital, 145 mM NaCl, 0.1% gelatin, pH 7.4. By adding MgCl_2_ and CaCl_2_ to the final concentrations of 0.5 mM and 0.15 mM, respectively, GVB+ was prepared; EGTA was added to the final concentrations of 5 mM to ensure Ca^+2^/Mg^+2^ depletion. wHS or complement component Factor-B-depleted serum (CompTech, TX, USA) was diluted in GVB+/- (8 µl serum + 92 µl GVB) and was added to the PRM-coated wells, incubated at ambient temperature for 1 h, washed twice with wash buffer, added with peroxidase-conjugated anti-human complement C3 antibody (MP Biomedicals; Cat-No. 55237) in PBS-BSA (1:5,000 dilution, 100 µl/well), and incubated at ambient temperature for 1 h. The wells were washed thrice with wash buffer and added with the substrate (TMBW, Tebu-Bio; 100 µl/well). After 5 min, the color development was arrested by adding 4% H_2_SO_4_ (50 µl/well). The optical density was measured at 450 nm using microplate reader (Infinite m200 pro, TECAN). C3-PRM interaction was also studied using C1q and MBL-depleted sera following the protocol described for wHS.

For PRM-CR3 interaction study, PRM-coated wells (50 µg/ml in sodium bicarbonate buffer) in a 96-well microtiter plate were added with recombinant human CD11b (50–100 ng/well; Abcam ab126010) in PBS-BSA (1%), incubated at ambient temperature for 1 h, washed with PBS-Tween-20 (0.05%), added sequentially with monoclonal anti-human CD11b antibodies (Sony, clone M1/70) and secondary peroxidase-conjugated human IgG, incubated at ambient temperature for each antibody added, and washed with PBS-Tween-20 between each step. Furthermore, chromogenic peroxidase substrate (TMBW, Tebu-Bio; 100 µl/well) was added, and the color developed was optically read at 450 nm.

### Characterization of the Peptidorhamnomannan by NMR

PRMs from *S. brasiliensis* (16.0 mg/ml) and *S. schenckii* (14.5 mg/ml) were obtained as described previously (36), dissolved in D_2_O (99.99%, Eurisotop, France) to be analyzed by NMR in natural ^13^C abundance. NMR experiments were performed at 25°C on an 18.8 T Avance Neo spectrometer (Bruker, Billerica, USA) with a ^1^H resonating frequency of 800.62 MHz equipped with a triple resonance (^1^H, ^15^N, ^13^C) cryogenically cooled probe. Spectra were recorded and processed with Topspin 4.08 (Bruker) and analyzed with CCPNMR Analysis 2.5 (37).

To assign the carbohydrate moiety resonances of PRMs, we analyzed ^1^H homonuclear 1Ds and 2D DQFCOSY [double-quantum-filtered correlation spectroscopy (38)], TOCSY [total correlation spectroscopy, 60 ms mixing time (39)], ROESY [rotating frame Overhauser enhancement spectroscopy, 200 ms mixing time (40)], and NOESY [nuclear Overhauser enhancement spectroscopy, 200 ms mixing time (41)] spectra and 2D ^1^H–^13^C heteronuclear experiments: HSQC [heteronuclear single quantum coherence (42)] and its edited version to differentiate CH_2_ groups from CH and CH_3_ groups, HMBC [heteronuclear multiple bond coherence (43), HSQCTOCSY (30, 60 and 100 ms mixing times] and H2BC [heteronuclear two bond correlation (44)]. Typically, the spectral width for ^1^H was 5.5 ppm (centered at 3.1 ppm) and 56 ppm for carbon (centered at 84 ppm). ^1^H–^13^C spectra were folded and recorded with non-uniform sampling (50%) in the indirect ^13^C dimension to increase resolution.

### Statistical Analysis

Statistical analyses were performed using GraphPad Prism 9 (San Diego, CA, USA). Unpaired two-tailed t-tests and one-way analysis of variance (ANOVA) with Tukey’s multiple comparison posttest or Dunnett’s multiple comparison posttest were performed. The statistical details of each result can be found in the figure legends; “n” represents the total number of replicates used in the assay and statistical analysis. Differences between data were considered significant when p ≤ 0.05.

## Results

### Phagocytosis of *Sporothrix* Species Yeast Cells Is Impaired by Thermolabile Serum Factors

**Figure 1:**
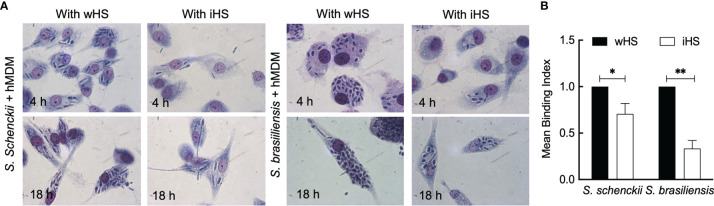
Interaction between the *Sporothrix* yeasts and human monocyte-derived macrophages (hMDMs). **(A)** *Sporothrix schenckii* or *Sporothrix brasiliensis* yeast cells were made to interact with hMDMs at 37°C in a CO_2_ incubator for 4–18 h in medium containing whole human serum (wHS) or heat-inactivated human serum (iHS). Optical photomicrographs were recorded at ×100 magnification; at least three biological replicates, each in duplicate, were performed. **(B)** hMDMs were infected with FITC-labeled yeasts of *S. schenckii* or *S. brasiliensis* in the presence of wHS or iHS for 1 h at 37°C in a CO_2_ incubator, washed with PBS, incubated for 15 min with Trypan blue, washed, and subjected to flow cytometry after gently releasing hMDMs from the wells using a cell scraper. Results are mean ± SD of three experiments (**p < 0.001 and *p < 0.05).

First, the ability and dynamics of hMDMs to recognize and phagocytose *S. schenckii* and *S. brasiliensis* yeasts were monitored. These human immune cells in the presence of autologous serum were able to actively phagocytose the yeasts of both *Sporothrix* species within 30 min of interaction (**Supplementary Videos S1** and **S2**). After 18 h of interaction of *Sporothrix* yeasts with hMDMs, some germinating cells could be observed (**Figure 1A**). Moreover, an increase in the number of *S. brasiliensis* yeasts inside the hMDMs after 18 h of interaction was noticed. The germination of *S. brasiliensis* inside hMDM is illustrated by a microscopic observation after an overnight interaction (18 h) in the presence of wHS (**Supplementary Video S3** **)**, which demonstrates the uptake of *S. brasiliensis* yeasts by hMDMs and their germination within these cells.

**Figure 2:**
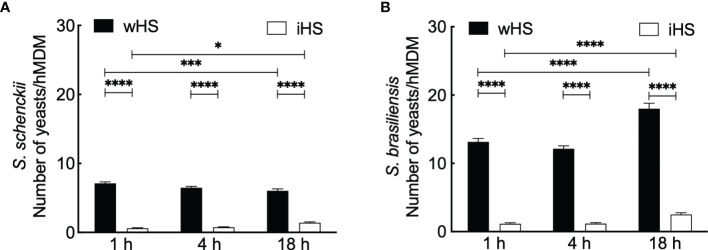
Kinetics of the interaction between *Sporothrix* and human monocyte-derived macrophages (hMDMs). **(A)** *Sporothrix schenckii* or **(B)** *Sporothrix brasiliensis* yeasts were made to interact with hMDMs at 37°C in a CO_2_ incubator for 1, 4, and 18 h in medium containing human serum [whole (wHS) or inactivated (iHS)]. Mean ± SEM of the yeasts internalized by hMDMs after indicated interaction period for which at least 10 optical microscopy fields were analyzed; a minimum of 50 hMDMs were counted for internalized yeasts; three biological replicates, in duplicate, were performed. Statistical analysis was performed by ANOVA with Tukey’s multiple comparison posttest (****p < 0.0001, ***p < 0.001, and *p < 0.01).

We then performed phagocytic studies in media supplemented with wHS or heat-inactivated human serum (iHS) (**Figure 1B**). After 1 h interaction with hMDMs, flow cytometry analysis showed a significant decrease in the uptake of the two *Sporothrix* species in the medium containing iHS compared to that containing wHS, suggesting that thermolabile serum factors facilitate the uptake of *Sporothrix* yeasts by hMDMs. On the other hand, the number of *Sporothrix* yeasts taken up by hMDMs was higher for *S. brasiliensis* compared to *S. schenckii* (**Figure 2**). Moreover, there was an increase in the number of *S. brasiliensis* yeast in hMDMs after 18 h (**Figure 2B**), consistent with the microscopic observation (**Figure 1**), suggesting their replication inside hMDMs. Together, these results confirm that hMDMs recognize *S. schenckii* and *S. brasiliensis* yeasts distinctively and that thermolabile serum factors mediate the uptake of both species, albeit at different degrees.

### *S. brasiliensis* Is Cytotoxic to Human Monocyte-Derived Macrophages

**Figure 3:**
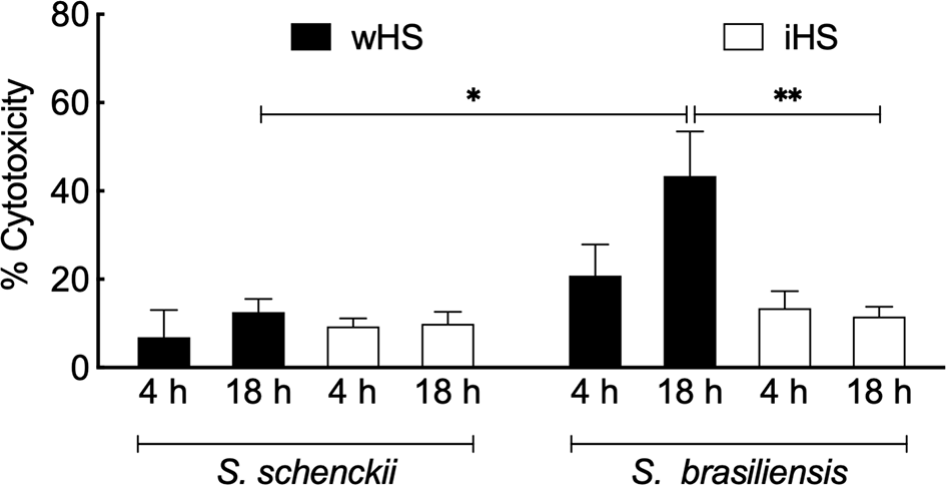
*Sporothrix* mediated cytotoxicity of human monocyte-derived macrophages (hMDMs)*. Sporothrix schenckii* or *Sporothrix brasiliensis* yeasts were incubated with hMDMs at 37°C in a CO_2_ incubator for 4–18 h in the medium containing whole (wHS) or inactivated (iHS) human serum. Following, lactate dehydrogenase (LDH) activity was measured using CytoTox^®^ Non-Radioactive Cytotoxicity Assay (Promega) kit. Results are the mean ± SEM values of two independent experiments in duplicate. Statistical analysis was performed by ANOVA with Turkey’s multiple comparison posttest (**p < 0.005 and *p < 0.05).

Cytotoxicity of *Sporothrix* species was determined by the LDH assay upon 4–18 h of interaction in hMDMs challenged with the *Sporothrix* yeasts. The results are expressed by the relative percentage to the uninfected control in **Figure 3**. In the medium containing iHS, both *Sporothrix* species showed less than 20% of cytotoxicity, which could be related to their low phagocytic levels. On the other hand, after 18 h of interaction in the presence of medium containing wHS, the cytotoxicity of *S. brasiliensis* was significantly higher compared to that of *S. schenckii* (p < 0.05), which could be attributed to the higher level of phagocytosis of *S. brasiliensis* and its capacity to germinate/multiply.

### *Sporothrix* Species Induce a Pro-Inflammatory Response by hMDM

**Figure 4:**
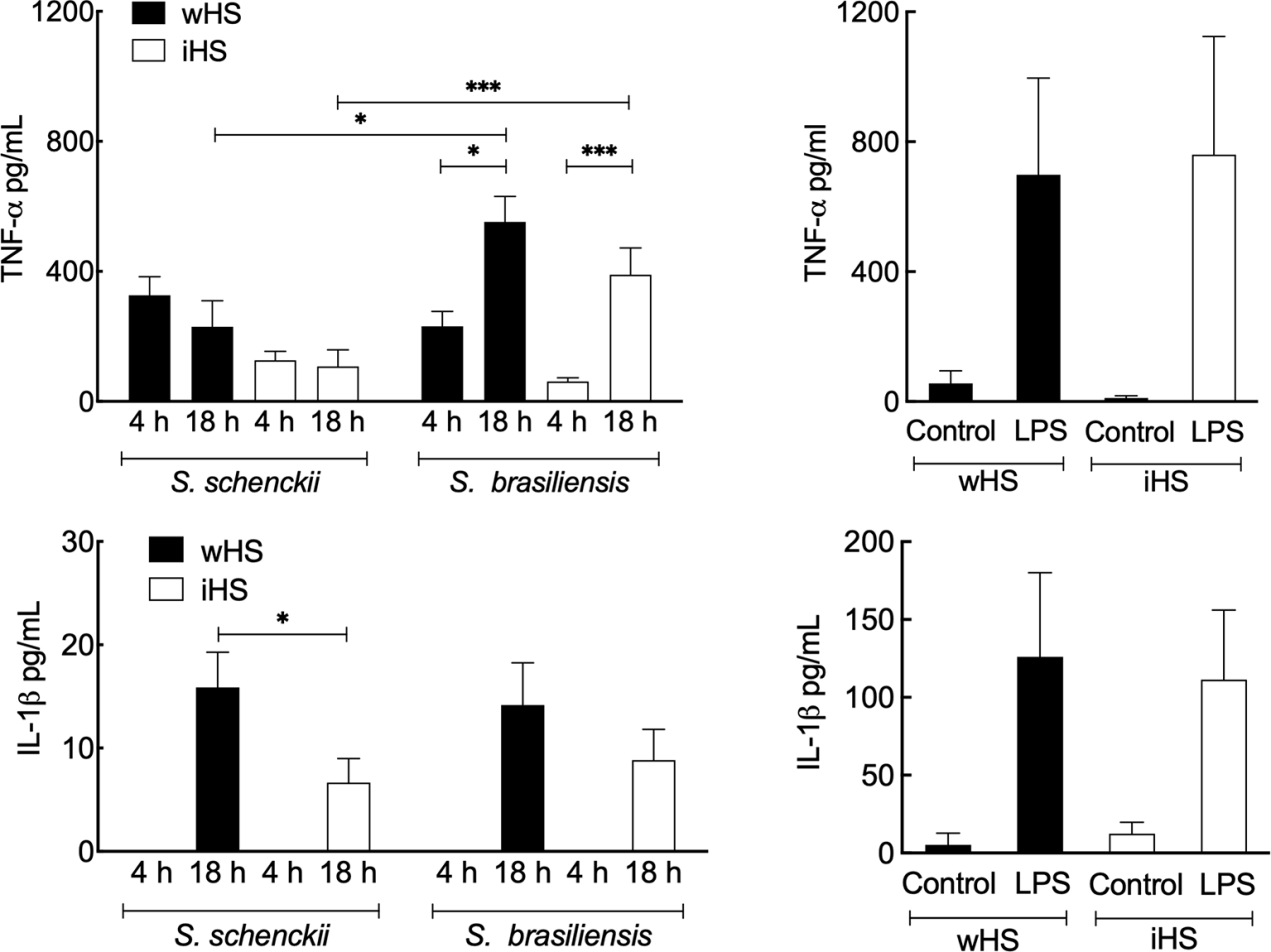
Cytokine release by human monocyte-derived macrophages (hMDMs) following interaction with *Sporothrix* species. *Sporothrix schenckii* and *Sporothrix brasiliensis* yeasts were made to interact with hMDMs in culture medium supplemented with human serum [whole (wHS) or inactivated (iHS)] for 4–18 h at 37°C and 5% CO_2_. The culture supernatants were collected and analyzed for tumor necrosis factor (TNF)-α and interleukin (IL)-1β [positive control was the lipopolysaccharide (LPS)-stimulated hMDM]. Results are the mean ± SEM of at least two independent experiments ran in duplicate (n = 4 or 6). Statistical analysis was performed by ANOVA with Tukey’s multiple comparison posttest (***p < 0.005 and *p < 0.05).

The secretion of the pro-inflammatory cytokines, TNF-α and IL-1β, was evaluated after 4 and 18 h of interaction of *Sporothrix* yeasts with hMDMs (**Figure 4**). In the medium containing wHS, both *S. schenckii* and *S. brasiliensis* stimulated the secretion of TNF-α by hMDMs at comparable levels, which remained at the same level for *S. schenckii* even after 18 h of interaction. However, from 4 to 18 h, there was a significant increase in the TNF-α secretion by hMDMs stimulated with *S. brasiliensis*. IL-1β was detectable in the *Sporothrix*–hMDM culture supernatant only after 18-h interaction. For the medium supplemented with iHS, lower levels of TNF-α and IL-1β secretion by hMDMs were observed for both *Sporothrix* species. Furthermore, only *S. brasiliensis*-stimulated hMDMs showed a significant increase in the TNF-α secretion during the course of incubation from 4 to 18 h. Neither of the *Sporothrix* species could stimulate a significant release of IL-10 in either wHS- or iHS-supplemented media. To rule out any artifact in the hMDM cytokine secretion profile due to any indirect effect of iHS compared to wHS, similar experiments were performed with LPS as a positive control. The hMDM cytokine secretion profile in response to LPS stimulation was similar in both wHS- and iHS-supplemented media (**Figure 4**). These results suggest that the hMDM cytokine response was not directly influenced by human serum supplement (wHS or iHS) in the culture medium, but through their effect on the phagocytosis of *Sporothrix* yeasts (**Figure 2**).

### The Complement System Is Involved in the Inflammatory Response of *Sporothrix* Species

**Figure 5:**
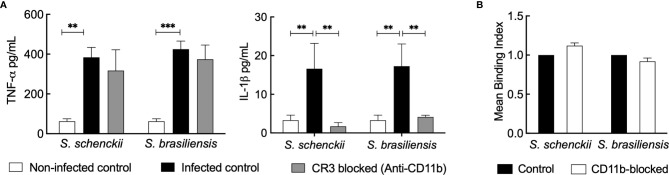
**(A)** Cytokine released by human monocyte-derived macrophages (hMDMs) blocked for complement receptor-3 (CR3) and challenged with *Sporothrix* species. *Sporothrix* yeasts were made to interact with hMDMs blocked for CR3 (with monoclonal anti-CD11b antibodies) or unblocked (infected control) in a culture medium supplemented with whole human serum (wHS) for 18 h at 37°C and 5% CO_2_. Non-infected controls were hMDMs blocked with irrelevant antibodies and treated with culture medium supplemented with wHS. The culture supernatants were collected and analyzed for tumor necrosis factor (TNF)-α and interleukin (IL)-1β. Results indicate the mean ± SEM of at least two independent experiments in duplicate or triplicate (n = 4 or 6). ANOVA with Tukey’s multiple comparison posttest was performed for the statistical analysis (***p < 0.001, **p < 0.001). **(B)** Uptake of *Sporothrix* yeasts by hMDMs after CR3 blockage. hMDMs were incubated with monoclonal anti-CD11b antibodies for 1 h and challenged with FITC-labeled yeasts of *Sporothrix schenckii* or *Sporothrix brasiliensis.* After 4 h of interaction in the presence of medium containing wHS, hMDMs were analyzed by FACS to determine the change in mean binding index (MBI) compared to control (=CD11b unblocked hMDMs interacted with FITC-labeled *Sporothrix* yeasts). Results are the mean ± SD of an experiment performed in duplicate.

As heat inactivation of the human serum directly impacts the phagocytosis and inflammatory response of hMDMs toward *Sporothrix* species, we investigated the role of the heat-labile complement system in the serum against these species. We first targeted CR3 (the major complement receptor expressed on macrophages) (45). Upon blocking CD11b and CD18, the CR3 components, using monoclonal antibodies, there was a significant reduction in the secretion of IL-1β by *Sporothrix*-infected hMDMs (**Figure 5A**). Nevertheless, the blockage of CD11b (the unique component of CR3) did not inhibit the uptake of *S. schenckii* and *S. brasiliensis* yeasts, suggesting the involvement or the cooperation of other pattern recognition receptors (PRRs) in their recognition and phagocytosis by hMDMs (**Figure 5B**).

**Figure 6:**
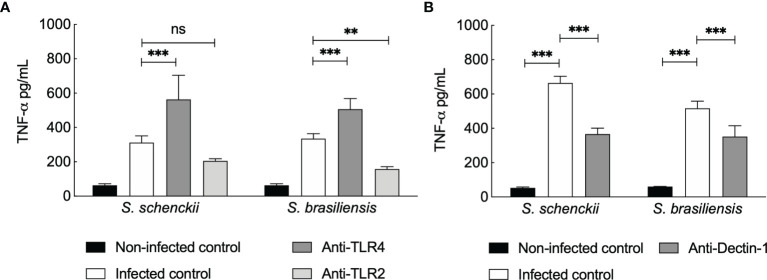
Tumor necrosis factor (TNF)-α secreted by *Sporothrix* species infected human monocyte-derived macrophages (hMDMs) blocked for Toll-like receptor (TLR)4 and TLR2 **(A)** and Dectin-1 **(B)**. TLRs and Dectin-1 on hMDMs were blocked by preincubation (1 h) with TLR2-, TLR4-, or Dectin-1-specific monoclonal antibodies, followed by interaction with *S. schenckii* or *S. brasiliensis* yeasts, in medium containing whole human serum (wHS). Non-infected controls were preincubated with medium containing wHS, and infected controls were incubated with *Sporothrix* species. Results are the mean ± SEM of at least two independent experiments performed in at least triplicate (n = 3 or more). Statistical analysis was performed by two-way ANOVA with Tukey’s multiple comparison posttest (ns = not significant; ****p < 0.0001, ***p < 0.001, and **p < 0.01).

As the TNF-α secretion by hMDMs upon *Sporothrix* species infection was not inhibited by blocking CR3, we further investigated the possible participation of other PRRs (**Figure 6**) already described to mediate TNF-α release by human PBMCs infected with *Sporothrix* species morphotypes (25). TLR4 blockage on hMDMs resulted in an increase in the production of TNF-α upon infection of hMDMs with *Sporothrix* species (**Figure 6A**). In contrast, a significant reduction in TNF-α secretion was observed upon TLR2 blockage of hMDMs challenged with *Sporothrix* species, although this reduction was not statistically significant for *S. schenckii*-infected hMDMs blocked for TLR2 (**Figure 6A**). Blockage of Dectin-1 on hMDMs also resulted in significant differences in the TNF-α secretion by hMDMs compared to unblocked hMDMs, suggesting that TNF-α secretion by hMDMs upon interaction with *Sporothrix* is also Dectin-1 recognition dependent (**Figure 6B**).

### Peptidorhamnomannans in the *Sporothrix* Cell Wall Activate Complement System

**Figure 7:**
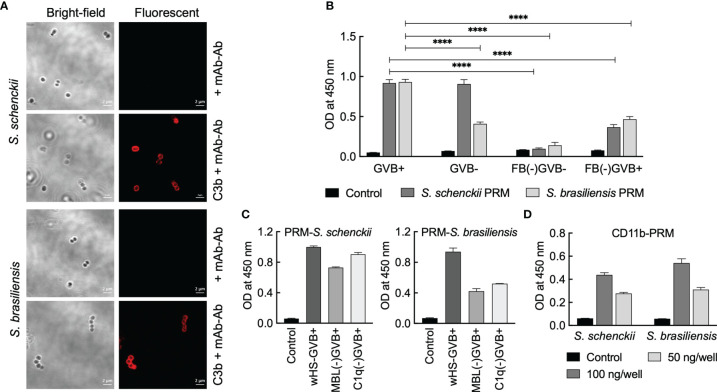
Interaction between complement system and peptidorhamnomannans (PRMs) of *Sporothrix* species. **(A)** *Sporothrix* yeasts were opsonized with C3b and labeled with mouse anti-C3b mAb and mouse TRITC-IgG (Ab). PRMs (50 µg/ml in 50 mM carbonate buffer, pH 9.6) were coated on 96-well plate: their **(B, C)** C3 activation capacities were determined using GVB+ or GVB- (with EGTA) media using whole/complement factor-depleted human sera. **(D**) Interaction with CD11b was determined by sequential addition of CD11b, mouse anti-CD11b mAb, peroxidase-conjugated mouse IgG, and chromogenic substrate for peroxidase (tetramethyl benzidine); colors developed were optically read at 450 nm. For each condition, two independent experiments, in triplicate, were performed. Statistical analysis was performed by ANOVA with Tukey’s multiple comparison posttest (****p < 0.0001).

We first checked the opsonization of *Sporothrix* yeasts with C3b. Immunolabeling indicated that C3b gets deposited on the surface of the yeasts of both *S. schenckii* and *S. brasiliensis* (**Figure 7A**). As the fungal cell wall components are the major source of PAMPs and the first to interact with the host immune system (22) and considering that the PRM is a well-characterized component located on the outermost cell wall layer of *S. schenckii* and *S. brasiliensis* yeast parasitic phase (31), we examined the complement activation capacity of the PRMs extracted from these two *Sporothrix* species. Total carbohydrate content in the PRMs of the two *Sporothrix* species was determined with phenol-sulfuric acid method to use them at equal concentrations for the investigation of their complement activation capacities. In GVB+ (containing Ca^+2^/Mg^+2^) medium, which facilitates the activation of all three complement (classical, lectin, and alternative) pathways, PRMs from both *S. schenckii* and *S. brasiliensis* could activate the complement system (readout was deposited C3b).

In GVB- medium (with EGTA, a Ca^+2^/Mg^+2^ chelator) that allows the activation of only the alternative pathway, PRM from *S. schenckii* showed C3b deposition similar to that in GVB+ medium, but there was a significant decrease in the complement activation by *S. brasiliensis* PRM. This suggests that the PRM of *S. schenckii* activates the complement system mainly *via* the alternative pathway, whereas that of *S. brasiliensis* partially activates complement system through alternative pathway. In accordance, complement Factor-B (FB)-depleted serum in GVB- medium completely abolished the complement activation by PRMs extracted from both *S. schenckii* and *S. brasiliensis* (**Figure 7B**), as FB is involved in the amplification loop for the activation of C3 into C3b *via* alternative pathway. On the other hand, FB-depleted serum in GVB+ medium allowed partial regaining of the complement activation by PRMs from both *Sporothrix* species (**Figure 7B**), suggesting that *S. brasiliensis* PRM is also capable of activating complement system through classical and lectin pathways. Furthermore, these observations are supported by mannose-binding lectin (MBL)- and C1q-depleted sera (the key components of lectin and classical pathways, respectively), wherein *S. schenckii* PRM was mostly independent of MBL/C1q for C3 activation and *S. brasiliensis* PRM showed partial dependence (**Figure 7C**).

We also investigated the direct interaction between PRMs and CD11b, the specific component of CR3. ELISA upon coating PRMs and incubating them with CD11b indicated that there is direct and specific interaction between PRMs from both *S. schenckii* and *S. brasiliensis*, as the binding between PRM and CD11b was concentration dependent (**Figure 7D**). Altogether, the results suggest that PRMs could be the PAMPs of *Sporothrix* species directly recognized by the CR3 expressed on hMDMs.

### Peptidorhamnomannans of the Two *Sporothrix* Species Are Structurally Different

**Figure 8:**
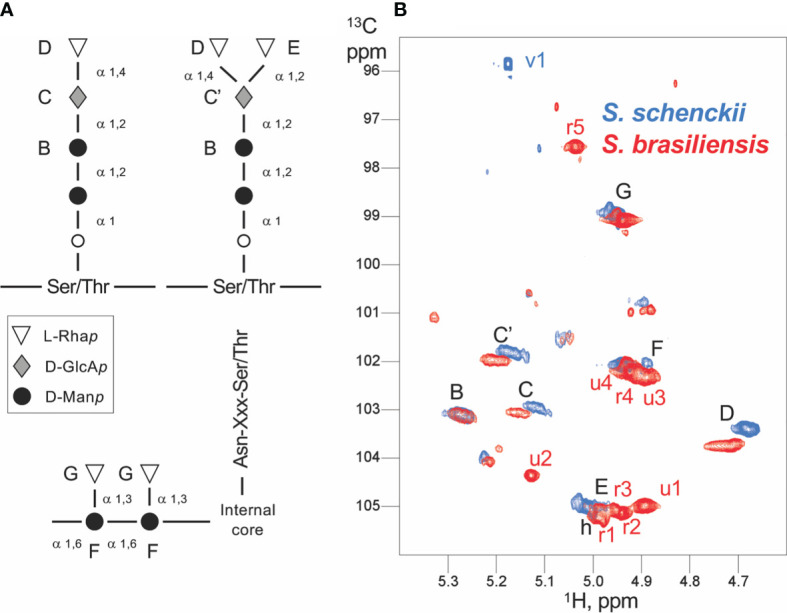
Scheme of the *Sporothrix* peptidorhamnomannan (PRM) organization and NMR assignments. **(A)** The generally known structures of the O-linked (top) and N-linked rhamnomannan (bottom) are shown. The inter-residue linkage types are displayed. The capital letters from B to G denote the assignments obtained by NMR, and the corresponding signals are displayed in panel **(B). (B)** Anomeric region of the ^1^H-^13^C HSQC spectra of *Sporothrix schenckii* (blue) and *Sporothrix brasiliensis* (red) crude PRMs. Each signal arises from the anomeric ^1^H-^13^C group of a distinct polysaccharide unit. The signals common to both species (and thus common residues) are labeled in black, whereas the signals unique to a species are labeled in the corresponding color. Capital letters denote signals from assigned residues; the corresponding residues are labeled in the PRM organization scheme **(A)** . The mannose O-linked to Ser/Thr could not be assigned. Whereas *S. schenckii* only shows a single residue absent (v1) in *S. brasiliensis*, the later displays at least nine unique residues with five identified rhamnose units (r1 to r5) and four yet unidentified units (u1–u4).

At this point, it was important to have structural data to test whether *S. brasiliensis* and *S. schenckii* PRMs were different, which could be at the origin of the differential complement activation pathways. Thus, to correlate the PRM structures with the different immune response behavior to *Sporothrix* species, we characterized *S. schenckii* and *S. brasiliensis* PRMs by ^1^H and ^1^H-^13^C NMR. Analysis of the 2D homonuclear and ^1^H-^13^C spectra of PRMs led to the assignment of many monosaccharide units and their linkages (**Figure 8**). As described earlier for *S schenckii* (46), *S. brasiliensis* PRM contains chains of linear Rha*p-*α (1,4) GlcA*p-*α (1,2) Man*p* α1. α-Rhamnose residues were additionally linked to disubstituted GlcA*p* through α (1,2) linkages (**Figure 8** **)**. These chains have previously been ascribed to O-linked rhamnomannans of *S. schenckii*, linked to Ser/Thr residues of the peptide moiety by an additional mannose residue (46, 47). Here, however, we could not assign the later peptide O-linked mannose. These Rha*p-*α (1,4) GlcA*p-*α (1,2) Man*p* α1 and Rha*p-*α (1,4) [Rha*p-*α (1,2)] GlcA*p-*α (1,2) Man*p* α1 structure is here described, for the first time, in the *S. brasiliensis* PRM. NMR data further indicated that *S. brasiliensis* displayed Man α (1,6) chains with α-Rha residues branched at position 3 (31), a type of chain that was previously ascribed to N-linked PRMs of *S. schenckii* (47, 48). Thus, PRMs of both the *Sporothrix* species share common O- and N-linked chains. Nevertheless, it is important to underline that *S. brasiliensis* contains at least nine additional sugar residues with respect to *S. schenckii* (**Figure 8**). Five of these were assigned to rhamnose, in agreement with the higher rhamnose content observed of *S. brasiliensis* (31). Despite common elements in both species PRMs, the *S. brasiliensis* PRMs contain additional rhamnose-rich structure(s).

### *Sporothrix*-Mediated Pentraxin 3 Secretion by Human Monocyte-Derived Macrophages Is Complement System Dependent

**Figure 9:**
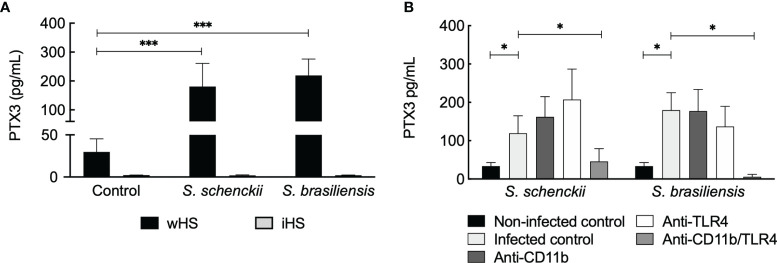
*Sporothrix schenckii* and *Sporothrix brasiliensis* yeasts stimulate pentraxin 3 (PTX3) secretion by human monocyte-derived macrophages (hMDMs). **(A)** Only in the medium supplemented with whole human serum (wHS), *S. schenckii* or *S. brasiliensis* yeasts stimulated the secretion of PTX3 by hMDMs but not in the medium containing inactivated human serum (iHS), suggesting that the thermolabile complement system plays a role during this process. Statistical analysis was performed by ANOVA with Tukey’s multiple comparison posttest (***p < 0.001). **(B)** Complement receptor-3 (CR3) crosstalks with Toll-like receptor (TLR)4 in stimulating PTX3 secretion by hMDMs upon interaction with *S. schenckii* or *S. brasiliensis* yeasts. Statistical analysis was performed by ANOVA with Tukey’s multiple comparison posttest (*p < 0.05).

The long PTX3 is a soluble pattern recognition molecule produced by somatic and immune cells at the site of infection. It is reported to crosstalk with other factors of the innate immune system (48). Our data showed an increase in PTX3 secretion by hMDMs challenged with *Sporothrix* species (**Figure 9A**). Interestingly, PTX3 secretion by hMDMs stimulated with *S. schenckii* and *S. brasiliensis* was evident only in the medium supplemented with wHS but not in the presence of iHS, suggesting the requirement of complement-mediated phagocytosis of *Sporothrix* species for the secretion of PTX3 by the immune cells. On the other hand, blockage of CR3 did not have any effect on PTX3 secretion by hMDMs upon interaction with *S. schenckii* and *S. brasiliensis* yeasts, whereas blockage of both CR3 and TLR4 (but not TLR4 alone) resulted in a significant decrease in the PTX3 secretion (**Figure 9B**). This crosstalk between the complement system and TLR4 in stimulating PTX3 production by hMDMs upon interaction with *S. schenckii* or *S. brasiliensis* and the role of PTX3 against *Sporothrix* species are subjects to be investigated further.

## Discussion

In this study, we describe the role played by (i) the host complement system in the recognition and *in vitro* response of hMDMs to two pathogenic species of the *Sporothrix* genus, *S. schenckii* and *S. brasiliensis*; (ii) the CR3 in mounting an inflammatory response toward *Sporothrix* species; and (iii) the cell wall component of *S. schenckii* and *S. brasiliensis*, the PRM, as the PAMP interacting with the complement system. *In vitro*, the level of phagocytosis of *S. brasiliensis* yeast by hMDMs in the presence of medium containing wHS was higher compared to *S. schenckii*. This observation is corroborated by our previous findings showing that, independently of the growth condition, *S. brasiliensis* yeasts were more phagocytosed by the human macrophages than the *S. schenckii* ones (31). Importantly, there was a significant decrease in the level of phagocytosis of both *S. schenckii* and *S. brasiliensis* by the hMDMs upon heat inactivation of the complement system. *S. brasiliensis* seems to be able to germinate inside the hMDMs after prolonged interaction times, suggesting that its higher survival capacity inside the hMDMs imparts more cytotoxicity and stimulates increased secretion of TNF-α compared to hMDMs challenged with *S. schenckii* in late interaction periods.

Intracellular infection of the macrophages and polymorphonuclear cells by *S. schenckii* is a typical histopathologic finding in human tissue lesions of sporotrichosis (49). Indeed, our initial observation was that, within 30 min, the hMDMs were able to phagocytose almost all yeasts of both *S. schenckii* and *S. brasiliensis* present in the interaction milieu (**Supplementary Material**). In our study, heat inactivation of serum drastically impaired the phagocytosis of both *S. schenckii* and *S. brasiliensis* by hMDMs, suggesting that thermolabile serum factors are central for the phagocytosis of *S. schenckii* and *S. brasiliensis*. Our findings support previous work showing that the human serum opsonins are crucial for the phagocytosis of *S. schenckii* yeasts by the THP-1 cells (50). It would be possible to argue that the phagocytosis by hMDMs and the consequent cytokine response could be simply impaired by any physiological reason due to culture conditions arising from the heat inactivation of the human serum. However, the inflammatory responses of hMDMs toward LPS in the culture medium containing whole (wHS) or heat-inactivated (iHS) human sera were similar, confirming that the reduced phagocytosis of *Sporothrix* species observed in the presence of iHS-supplemented media was indeed due to the lack of heat-labile serum factors.

It has been documented that the innate immunity plays an essential role in establishing a protective anti-*Sporothrix* response (23, 24, 51–54). In the last two decades, progress has been made to identify the PRRs involved in the innate immune response against *Sporothrix*. However, as most of these studies were focused on *S. schenckii*, the innate immune response remained unknown for *S. brasiliensis* and the PAMPs involved in the recognition of both *Sporothrix* species needed to be characterized. A recent work of our group showed that *S. schenckii* and *S. brasiliensis* present differences in their cell wall structure and composition, indicating a 100% higher rhamnose content on the cell wall PRM of *S. brasiliensis* (31). Moreover, *S. brasiliensis* exhibited a 400-nm external fibrillar layer composed of PRM and dissimilar to the tiny fibrils observed on *S. schenckii* cell surface. A specific ^1^H NMR signal exclusive of *S. brasiliensis* PRM and absent in *S. schenckii* PRM drove our attention to possible structural differences (31). Altogether, these observations suggested that *S. brasiliensis* PRM could exhibit undescribed chemical residues that may impair the host innate immune response. Another recent work correlated the host immune response with NMR data showing unique structures in the cell wall mannoprotein of *Candida auris* not found in the *Candida albicans* mannoproteins (55). In the last decade, a growing importance has been given to the impact of structural data on specific cell wall glycoconjugates present on the surface of fungal pathogens and the host immune response to each genus and/or species (22). These studies require an overlap of biochemical and ultrastructural data of a cell wall component and knowledge on the crosstalk with specific PRRs of host cells and its impact on the host immune response (22, 55–58). The fungal cell wall is composed of 80% sugars and, in general, the glycoproteins are present on the outermost microfibrillar layer of the cell wall of yeasts as proposed for *Candida albicans* (59). The mannan structure in mannoproteins of *Candida* species is the best known among fungal pathogens (60). In contrast, rhamnomannans are unique structures found on the cell wall of *Sporothrix* and *Scedosporium* species (31, 46, 58), and data on their role on the host immune response are still scarce (58).

The cell wall plasticity among species of the same genus and how these spatial organizations due to unique residues present in a cell wall glycoconjugate impair the host interaction gain more biological importance. In this study, we show that PRMs from *S. schenckii* and *S. brasiliensis* share common structures and rhamnose residues (Rha 1-3 Man; Rha1-4GlcA and Rha 1-2 GlcA) that have been previously described for *S. schenckii* (46), but *S. brasiliensis* contains five unique rhamnose residues that are not present in the *S. schenckii* PRM as shown by the ^1^H and ^13^C NMR spectra. These five rhamnose residues do not match with any known *S. schenckii* rhamnomannan structure (47, 61), indicating the presence of new unknown rhamnose epitopes in the *S. brasiliensis* cell wall PRM. The differences in the cell wall architecture and PRM structure of *S. schenckii* and *S. brasiliensis* could explain the differences observed in the phagocytosis and host macrophage response toward each species. Moreover, the differences in PRM structure could be at the origin of the differential activation of complement pathways elicited by the PRMs of *S. schenckii* and *S. brasiliensis*.

The complement system contains heat-labile serum factors (62), and the major opsonins are the complement C3-derived fragments, namely, C3b, iC3b, and C3d, responsible for the facilitation of phagocytosis through complement receptors (63). Our study shows that C3b opsonizes yeasts of both *S. schenckii* and *S. brasiliensis.* CR3 has been shown to contribute to fungal recognition by the innate immune cells (64). CR3 is an enigmatic receptor, which transduces diverse and distinct signals upon engagement with different ligands (65, 66). However, the role of CR3 in the context of *Sporothrix* infection had never been studied. In this work, we show that the PRMs of both *S. schenckii* and *S. brasiliensis* are directly recognized by CR3; however, blockage of CR3 alone did not have any impact on the level of phagocytosis of both *S. schenckii* and *S. brasiliensis*, suggesting that there are other receptors/mechanisms involved in the phagocytosis of both species. Interestingly, however, CR3 blockage resulted in a total block of IL-1β secretion by hMDMs in response to *Sporothrix* stimulation. CR3 inside-out activation can be initiated from other receptors including TLRs and Dectin-1 (67, 68). The outside-in signal, on the other hand, can be driven by the engagement of CR3, which activates innate immune effector functions, such as phagocytosis, cytotoxic killing, and cytokine production (69). Our ongoing investigation, may clarify these questions.

Previous reports have shown that the absence of TLR2 causes an impaired phagocytosis, microbicide mechanisms (production of NO), and the cytokine secretion (TNF-α, IL-6, and IL-10) in *in vitro S. brasiliensis* infection (27). It was also demonstrated that the absence of TLR2 during experimental *S. brasiliensis* infection promoted increased dissemination after 14–28 days, suggesting a polarized Th17 response in an attempt to control the infection. However, our results demonstrate that TLR2 inhibition impacts only TNF-α secretion by *S. brasiliensis*-infected hMDMs. TLR4 is another receptor described as central in the induction of protective immune response to *S. schenckii* and *S. brasiliensis* (23–25, 27); it has indeed been described as a key receptor in the pro- and anti-inflammatory response of murine macrophages isolated from infected mice. On the contrary, our data did not indicate a key role of TLR4 in the inflammatory response of hMDMs toward *Sporothrix* infection. Moreover, blockage of Dectin-1 also had an impact on the TNF-α secretion by hMDMs, suggesting that *Sporothrix* yeasts are indeed recognized by Dectin-1, consistent with our earlier observation (25). Intriguingly, our results indicate that a soluble receptor, PTX3, is secreted by hMDMs challenged by both *Sporothrix* species. PTX3 has been reported to recognize other fungal pathogens (49); PTX3 facilitates the deposition of C1q, a key complement component involved in the activation of classical pathway of the complement system, thus enhancing the clearance of pathogens. Our data bring evidence that the secretion of PTX3 is impaired by the inactivation of human serum and that the complement system plays a crucial role for the PTX3 secretion by hMDMs challenged with *Sporothrix* species. However, blockage of both CR3 and TLR4 resulted in a significant decrease in the PTX3 secretion by hMDMs upon interaction with the *Sporothrix* species, suggesting a crosstalk between the complement system and TLR4, in accordance with the earlier report that TLRs crosstalk with the complement system (70). The mechanisms involving PTX3 and complement factors related to the physiopathology of sporotrichosis need to be investigated further. On the other hand, a recent review suggests that although synthesis of cytokines, their intracellular trafficking, and secretion are in response to activation of the PRRs or inflammasomes, there is no direct evidence linking the event of phagocytosis and cytokine product, explaining the involvement of multiple receptors for the secretion of different cytokines by hMDMs upon interaction with the *Sporothrix* yeasts, which needs to be explored further (71).

Decades ago, it was established that *S. schenckii* yeast cells can stimulate both the classic and alternative complement pathways (72, 73), although the specific molecules responsible for this process were still unknown. *Sporothrix* species present some particularities regarding their cell wall structure, with the presence of a PRM (31, 36, 47, 61). PRM is an integral component of both *S. schenckii* and *S. brasiliensis* cell walls present in the outermost fibrillar layer (31). We here studied the complement pathway-activating capacities of PRMs isolated from the yeast cells (parasitic morphotype) of both species. PRMs of both species could convert complement C3 into its activated form (C3b). When the assay was performed in the presence of medium containing a divalent ion chelator, which allows the alternative pathway of the complement system, there was no significant decrease in the complement-activating capacities of the PRM from *S. schenckii*. This suggested that *S. schenckii* rhamnomannans mainly elicit the alternative pathway of complement activation. In agreement, when complement Factor-B-depleted serum was used (devoid of alternative pathway), there was a significant decrease in the C3 activation by *S. schenckii* PRM. On the other hand, in the presence of medium containing a divalent ion chelator, PRM from *S. brasiliensis* showed a significant decrease in the complement activation, suggesting that the *S. brasiliensis* PRM only partially activates the alternative pathway. In addition, Factor-B-depleted serum in the absence of divalent cations almost completely abolished complement activation capacity, confirming that the complement activation by *S. brasiliensis* PRM occurs partially by the classical/lectin and alternative pathways.

In conclusion, the serum complement factors and complement activation by the cell wall PRMs are key players in the inflammatory response of hMDMs to *S. schenckii* and *S. brasiliensis*. We hypothesize that the process is initiated by the opsonization of the yeasts of these two *Sporothrix* species by heat-labile complement factors present in the human serum that facilitates the process of phagocytosis leading to CR3 inside-out activation. The yeasts opsonized by the complement factors would then elicit an inflammatory response *via* recognition by the CR3, and the TLR engagement could have a role in the regulation of macrophage inflammatory response. Our work contributes the new knowledge of PRM as a well-characterized PAMP that mediates the human macrophage inflammatory response toward *Sporothrix* species *via* the CR3 receptors expressed on the macrophages.

## Data Availability Statement

The original contributions presented in the study are included in the article/**Supplementary Material**. Further inquiries can be directed to the corresponding author.

## Ethics Statement

The studies involving human participants were reviewed and approved by Brazilian Health Ministry system, CEP/CONEP. The number of the Certificate of Presentation for Ethical Consideration related to this study is 62785716.2.0000.5259. The patients/participants provided their written informed consent to participate in this study.

## Author Contributions

LL-B, GB, and VA contributed to the conception of the study. NG, GB, CM, VA, and LL-B contributed to the design of the study. GN, SW, CW, JW, CS, and JG performed the experiments. GN, SW, VA, JG, CS, and LL-B contributed to data analysis. GN and SW performed statistical analysis. GN, VA, and JG performed figure editing. LL-B, VA, JG, and GB wrote the article. All authors contributed to the article and approved the submitted version.

## Funding

This work was supported by Fundação de Apoio à Pesquisa do Distrito Federal (FAP-DF)/CNPq, PRONEX grant ID: FAP-DF, 0193.001.200/2016. VA is supported by the Centre Franco-Indien pour la Promotion de la Recherche Avancée (CEFIPRA) grant no. 5403-1 and ANR-DFG AfuINF grant. JG, VA, and CS were supported by the ANR-FUNHYDRO (ANR-16S-CE110020-01) grant. NG, GB, and JW are supported by the Welcome Trust (102705, 097377, 101873, 215599, and 200208) and the Medical Research Council Centre for Medical Mycology (MR/N006364/2).

## Conflict of Interest

The authors declare that the research was conducted in the absence of any commercial or financial relationships that could be construed as a potential conflict of interest.

## Publisher’s Note

All claims expressed in this article are solely those of the authors and do not necessarily represent those of their affiliated organizations, or those of the publisher, the editors and the reviewers. Any product that may be evaluated in this article, or claim that may be made by its manufacturer, is not guaranteed or endorsed by the publisher.

## Supplementary Material

Supplementary Video 1 and 2 Live-cell video microscopy *Sporothrix* species phagocytosis assay with human monocyte-derived macrophages for up to 30 min. Human monocytes-derived macrophages (hMDM) were stained using the red fluorescent dye LysoTracker red DND-99, and *S. brasiliensis (ATCC 4824) yeasts were* stained using FITC (green). Following, hMDM and *S. brasiliensis* were co-cultured at 37°C in complete medium supplemented with 10% whole human serum. Images were captured at 1 min intervals and snapshots were taken from the video up to 30 minutes of interaction. Macrophages can be seen establishing cell-cell contact with *S. schenckii* (**Video S1**) and *S. brasiliensis* (**Video S2**), which is then followed by their uptake into macrophage. Click here to view movie 1/click here to view movie 2.

Supplementary Video 3 Live-cell video microscopy phagocytosis assay of *S. brasiliensis* with human monocyte-derived macrophages for up over 18 hours. Human monocytes-derived macrophages (hMDM) were stained using the red fluorescent dye LysoTracker red DND-99, and *S. brasiliensis (ATCC 4824) yeasts were* stained using FITC (green). Macrophages and *S. brasiliensis* were co-cultured at 37°C in complete medium supplemented with 10% whole human serum. Images were captured at 1 min intervals and snapshots were taken from the video up to 18 h of interaction. *S. brasiliensis* may survive and filament inside the hMDM. Click here to view movie.
